# Endoplasmic reticulum stress associated with lead (Pb)‐induced olfactory epithelium toxicity in an olfactory dark basal cell line

**DOI:** 10.1002/2211-5463.13714

**Published:** 2023-11-20

**Authors:** Bing Han, Teru Kamogashira, Shu Kikuta, Tatsuya Yamasoba

**Affiliations:** ^1^ Department of Otolaryngology and Head and Neck Surgery, Faculty of Medicine University of Tokyo Tokyo Japan; ^2^ Department of Otolaryngology and Head and Neck Surgery Nihon University Tokyo Japan

**Keywords:** dark basal cell, ER stress, lead, necroptosis, olfactory cell, Pb

## Abstract

Lead (Pb) can damage organs and also have undesirable effects on neural development. To explore the effects of Pb on olfactory cells, we investigated Pb‐induced cell toxicity in the DBC1.2 olfactory cell line, with a focus on endoplasmic reticulum (ER) stress, apoptosis, and necroptosis. Representative markers of ER stress, apoptosis, and necroptosis were analyzed by quantitative PCR. The mRNA expression levels of GRP94, GRP78, spliced XBP1, PERK, and ATF6 increased significantly after Pb exposure in a dose‐dependent manner. The expression of Caspase 3 and Caspase 12 did not increase after Pb exposure, which suggested that apoptosis‐induced cell death was not activated after Pb exposure. However, the mRNA of RIPK3 and MLKL showed increases in expression, which indicated that necroptosis‐induced cell death was activated after Pb exposure. These results indicate that Pb exposure induced dose‐dependent cytotoxicity through ER stress and necroptosis pathways in DBC1.2 cells, whereas the apoptosis pathway was not significantly stimulated. HEPES buffer showed a partial protective effect in terms of ER stress, apoptosis, and necroptosis. In summary, the necroptosis pathway plays a crucial rule in Pb exposure‐induced cytotoxicity in olfactory cells.

## Graphical abstract

In order to explore the harmful effects of lead (Pb) on the olfactory system, we investigated lead‐induced cell toxicity in the DBC1.2 olfactory cell line and analyzed markers of endoplasmic reticulum (ER) stress, apoptosis, and necroptosis; we found lead induced dose‐dependent cytotoxicity through ER stress and necroptosis pathways, and apoptosis was not significantly stimulated.

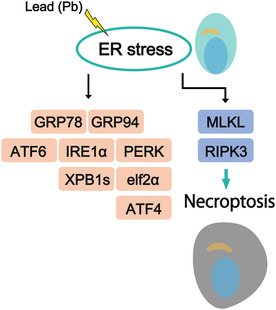

**Abbreviations**
10% FBS: 10% fetal bovine serum
ATF4: activating transcription factor 4
ATF6: activating transcription factor 6
CK14: cytokeratin 14
DBC1.2: dark basal cell 1.2
eIF2α: eukaryotic initiation factor 2α
ER stress: endoplasmic reticulum stress
GRP78: glucose regulating protein 78
GRP94: glucose regulating protein 94
IRE1α: inositol‐requiring enzyme 1α
MLKL: mixed lineage kinase domain‐like protein
PERK: protein kinase R (PKR)‐like endoplasmic reticulum kinase
RIPK: receptor‐interacting protein (RIP) kinases
TUBB3: class III β‐tubulin
XBP1: X‐box binding protein 1

Heavy metals including lead (Pb), chromium, cadmium, mercury, and arsenic can contaminate drinking water in the environment and pose a serious threat to human health [1, 2]. Lead exposure damages many organs [3, 4], and its effects prenatally are also critical especially in neural development [5]. There is no safe level of Pb exposure for the human body, even at low‐level Pb exposure is harmful [6, 7].

There are multiple pathways of Pb‐induced toxicity: (a) the reduction in the intracellular antioxidant system in hepatocytes [8] or in blood [9]; (b) the generation of reactive oxygen species (ROS), which can promote DNA damage [10, 11]; (c) inflammation in hepatocytes [12, 13], in lung [14], or in plasma [15]; (d) mitochondrial dysfunction, which leads to oxidative stress [16]; or (e) autophagy activated by endoplasmic reticulum (ER) stress, which can play a regulatory role in upstream mitophagy [17].

In the ER stress pathway, the misfolded proteins induced by toxic agents are detected by binding immunoglobulin protein (BiP)/glucose regulating protein 78 (GRP78) and multiple axes [18], which are protein kinase R (PKR)‐like endoplasmic reticulum kinase (PERK)‐eukaryotic initiation factor 2α (eIF2α)‐activating transcription factor 4 (ATF4) pathway, inositol‐requiring enzyme 1α (IRE1α)‐X‐box binding protein 1 (XBP1) pathway [19], and activating transcription factor 6 (ATF6) pathway, are stimulated, which followed by the stimulation of autophagy or the activation of apoptosis of cells [20, 21]. These ER stress pathways are also activated by necroptosis [22]. The difference of these multiple axes depends on the type of cells and is important for the targeting of therapeutics [23].

Programmed cell death includes several types of pathways, including apoptosis, necroptosis, and pyroptosis [24, 25]. Apoptosis plays a major role in developing and mature olfactory tissue [26, 27], and the renewal of the olfactory epithelium (OE) is related to the neuronal plasticity, which facilitates the detection of environmental odorants [28]. Recently, the role of necroptosis in addition to apoptosis in olfactory tissue was recognized as an important cell death pathway, and that necroptosis may facilitate the production and release of a myriad of proinflammatory cytokines and cause neutrophil infiltration in chronic rhinosinusitis with nasal polyps [29].

In necroptosis, two members of the receptor‐interacting protein kinase (RIPK) family, RIPK1 and RIPK3, are activated to phosphorylate mixed lineage kinase domain‐like protein (MLKL), which compromises the cell membrane to execute cell death. Apoptosis and necrosis can occur simultaneously because the signaling pathways interconnect with each other [30, 31]. The detection of the dynamic response of cell death pathways to toxic agents requires individual analysis.

Dark basal cells 1.2 (DBC1.2) is a cell line derived from DBC, which are also called horizontal basal cells (HBC), derived from a primary culture of OE of newborn mouse from embryonic day 14.5 [32]. The DBC1.2 cell line shows a Cytokeratin 14‐positive and neuron‐specific tubulin‐positive phenotype, and shares the properties of the DBC and neural cells [33]. This cell line can also be to evaluate drug agents in olfactory toxicity.

In this study, we investigated the pathways in which Pb induces cell toxicity in DBC1.2 olfactory cells from the aspect of apoptosis, necroptosis, and ER stress.

## Materials and methods

### Cell culture and culture conditions

DBC1.2 cells were purchased from the National Institutes of Biomedical Innovation, Health and Nutrition, Japanese Collection of Research Bioresources (JCRB) Cell Bank. The cells were either cultured in the original formulation [32] or a modified formulation. The original formulation was MCDB153 (IFP0010; Research Institute for the Functional Peptides Co., Ltd, Yamagata, Japan) supplemented with 0.1 mm ethanolamine (23406‐32; Nacalai Tesque, Kyoto, Japan), 0.1 mm phosphoethanolamine (27803‐31; Nacalai Tesque, Kyoto, Japan), 1.4 μg·mL^−1^ hydrocortisone (Solu‐Cortef for Intravenous Use; Pfizer, Tokyo, Japan), 10 ng·mL^−1^ mouse epidermal growth factor (050‐09101; FUJIFILM Wako Pure Chemical Corporation, Osaka, Japan), 5 ng·mL^−1^ recombinant human basic fibroblast growth factor (064‐05381; FUJIFILM Wako Pure Chemical Corporation, Osaka, Japan), 5 μg·mL^−1^ human insulin (099‐06473; FUJIFILM Wako Pure Chemical Corporation, Osaka, Japan), 10 μg·mL^−1^ bovine pituitary extract (13028014; Thermo Fisher Scientific, Waltham, MA, USA), 50 μg·mL^−1^ transferrin (from bovine plasma; FUJIFILM Wako Pure Chemical Corporation, Osaka, Japan), and 50 μg·mL^−1^ kanamycin (11981‐04; Nacalai Tesque, Kyoto, Japan). The modified formulation was Dulbecco's modified Eagle medium/nutrient mixture F‐12 (DMEM/F‐12) (08460‐95; Nacalai Tesque, Kyoto, Japan, 042‐30555; FUJIFILM Wako Pure Chemical Corporation, Osaka, Japan) containing 10% fetal bovine serum (FBS) (26140079; Life Technologies, Inc, Grand Island, NY, USA) and 100 units·mL^−1^ penicillin‐100 μg·mL^−1^ streptomycin (168‐23191; FUJIFILM Wako Pure Chemical Corporation, Osaka, Japan). Cells were incubated at 37 °C with 5% CO_2_.

### Immunohistochemistry preparation

The cells were fixed with 4% paraformaldehyde in phosphate‐buffered saline (PBS; pH 7.4; 10010023; Thermo Fisher Scientific Inc., Waltham, MA, USA) for 3 min and then washed three times with PBS. The cells were blocked with 0.3% Triton X‐100 and 5% bovine serum albumin (BSA) for 10 min and stained with CK14 (10143‐1‐AP; Proteintech, Rosemont, IL, USA) (1 : 500) and TUBB3 (B249869; Biolegend, San Diego, CA, USA) (1 : 500) in 0.3% Triton X‐100 and 5% BSA overnight at 4 °C. The next day, after washing three times with PBS, the tissues were stained with anti‐mouse IgG (Alexa Flour 594 A21207; Thermo Fisher Scientific, Waltham, MA, USA) (1 : 200) and anti‐rabbit IgG (Alexa Fluor 488 A11008; Thermo Fisher Scientific, Waltham, MA, USA) (1 : 200) in 0.3% Triton X‐100 and 5% BSA for 1 h at room temperature. VECTASHIELD Mounting Medium with DAPI (H‐1200; Vector Laboratories, Newark, CA, USA) was used for the embedding after washing three times with PBS. The fluorescent images were collected using a confocal microscope system (A1R; Nikon, Tokyo, Japan) with 60× (NA 0.95) Plan‐Apo lens.

### Pb exposure

HEPES buffer [34] was used to constitute a Pb‐containing exposure buffer, and lead (II) nitrate (129‐03222; FUJIFILM Wako Pure Chemical Corporation) was dissolved in HEPES buffer solution. The composition of HEPES buffer was modified based on HEPES‐Ringer (Krebs‐Ringer solution with HEPES) at the following concentrations: NaCl 140 mm, KCl 5 mm, CaCl_2_ 2 mm, MgCl_2_ 1 mm, HEPES 5 mm, and d‐glucose 10 mm.

### Cell viability assay and population analysis

Cell viability assay was performed using the Cell Counting Kit‐8 (CK04; Dojindo, Kumamoto, Japan) by incubating cells with WST‐8 for 1 h as directed by the manufacturer and measuring the absorbance at 450 nm using the infinite M200 PRO plate‐reader (TECAN, Zurich, Switzerland).

The number of cells was counted as follows: The cells in the plate were washed once with PBS, fixed with 4% paraformaldehyde (37152‐64; Nacalai Tesque, Kyoto, Japan) for 1 min, stained with Hoechst 33342 (final 5 μg·mL^−1^; Thermo Fisher Scientific, Waltham, MA, USA) for 30 min, washed once again with PBS, then captured with a fluorescence microscope (BZ‐X710; KEYENCE, Osaka, Japan) using the DAPI filter cube, and counted automatically using imagej [35, 36] with fiji [37].

### Quantitative PCR analysis

Total RNA was isolated with NucleoSpin RNA/Protein (740933.50; Macherey‐Nagel, Düren, Germany) according to the manufacturer's instructions. The concentration of extracted mRNA was measured using NanoDrop Lite (Thermo Fisher Scientific), and complementary DNA (cDNA) was generated using ReverTraAce qPCR RT Master Mix with gDNA Remover (FSQ‐301; Toyobo, Japan) according to the manufacturer's instructions. The quality of mRNA was assessed by visualization of the 28S and 18S ribosomal RNA bands using DynaMarker, RNA High for Easy Electrophoresis (DM170; BioDynamics Laboratory Inc., Japan). The primers and probes of genes including: Cytokeratin 14 (CK14) (forward: AGCGGCAAGAGTGAGATTTCT, reverse CCTCCAGGTTATTCTCCAGGG, final concentration: 250 nm), class III β‐tubulin (TUBB3) (forward: AGGTGCGTGAGGAGTACCC, reverse AGGGCTTCATTGTCGATGCAG, final concentration: 500 nm), receptor‐interacting protein kinase (RIPK1) (forward: GACAGACCTAGACAGCGGAG, reverse CCAGTAGCTTCACCACTCGAC, final concentration: 500 nm), RIPK3 (forward: GGCACCCTAGCGTACTTGG, reverse: GCTGTAGACATCACTCGCTTT, final concentration: 500 nm), mixed lineage kinase domain‐like protein (MLKL) (forward: GCCGGAGGCTACCAAGTAAAG, reverse: GTGGCAATTTCCCAGAGTACA, final concentration: 1000 nm), GRP78/BiP/Hsp5 (Heat Shock Protein Family A (Hsp70) Member 5) (forward: ACTTGGGGACCACCTATTCCT, reverse: ATCGCCAATCAGACGCTCC, final concentration: 250 nm), GRP94 (forward: TCGTCAGAGCTGATGATGAAGT, reverse GCGTTTAACCCATCCAACTGAAT, final concentration: 250 nm), IRE1α (forward: GCCGAAGTTCAGATGGAATC, reverse: ATCAGCAAAGGCCGATGA, final concentration: 1000 nm), XBP1 unspliced form (forward: GAATGGACACGCTGGATCCT, reverse: GCCACCAGCCTTACTCCACTC, final concentration: 500 nm), XBP1 spliced form (forward: GAGTCCGCAGCAGGTG, reverse: GTGTCAGAGTCCATGGGA final concentration: 1000 nm) [38], ATF6 (forward: CGGTCCACAGACTCGTGTTC, reverse: GCTGTCGCCATATAAGGAAAGG, final concentration: 500 nm), PERK (forward: AGTCCCTGCTCGAATCTTCCT, reverse: TCCCAAGGCAGAACAGATATACC, final concentration: 250 nm), Caspase 3 (forward: TGGTGATGAAGGGGTCATTTATG, reverse: TTCGGCTTTCCAGTCAGACTC, final concentration: 500 nm), Caspase 8 (forward: ACAAACCTCGGGGATACTGTC, reverse: AGTGCAGTCGTCGTAAGATACTA, final concentration: 500 nm), Caspase 12 (forward: GAAGGAATCTGTGGGGTGAA, reverse: TCAGCAGTGGCTATCCCTTT, final concentration: 500 nm), and GAPDH (forward: TGAGGCCGGTGCTGAGTATGTCG, reverse: CCACAGTCTTCTGGGTGGCAGTG, final concentration: 500 nm) were purchased from FASMAC (Kanagawa, Japan). The forward and reverse primers were mixed with 100 ng cDNA, and quantitative PCR was performed using THUNDERBIRD SYBR qPCR Mix (QPS‐201; Toyobo). The following experimental run protocol was used: denaturation and activation program (95 °C for 60 s), amplification and quantification program repeated 60 times (95 °C for 15 s, 60 °C for 45 s), and melting curve program (95 °C for 15 s, 60 °C for 15 s, 95 °C for 15 s). Data collection was performed using the QuantStudio 7 Flex Detection System (Thermo Fisher Scientific). The $2^{-\Delta \Delta C_{t}}$ method was applied to analyze the relative changes in gene expression. The mRNA expression level of each gene was normalized using GAPDH as an internal control.

### Statistical analysis

**Fig. 1:**
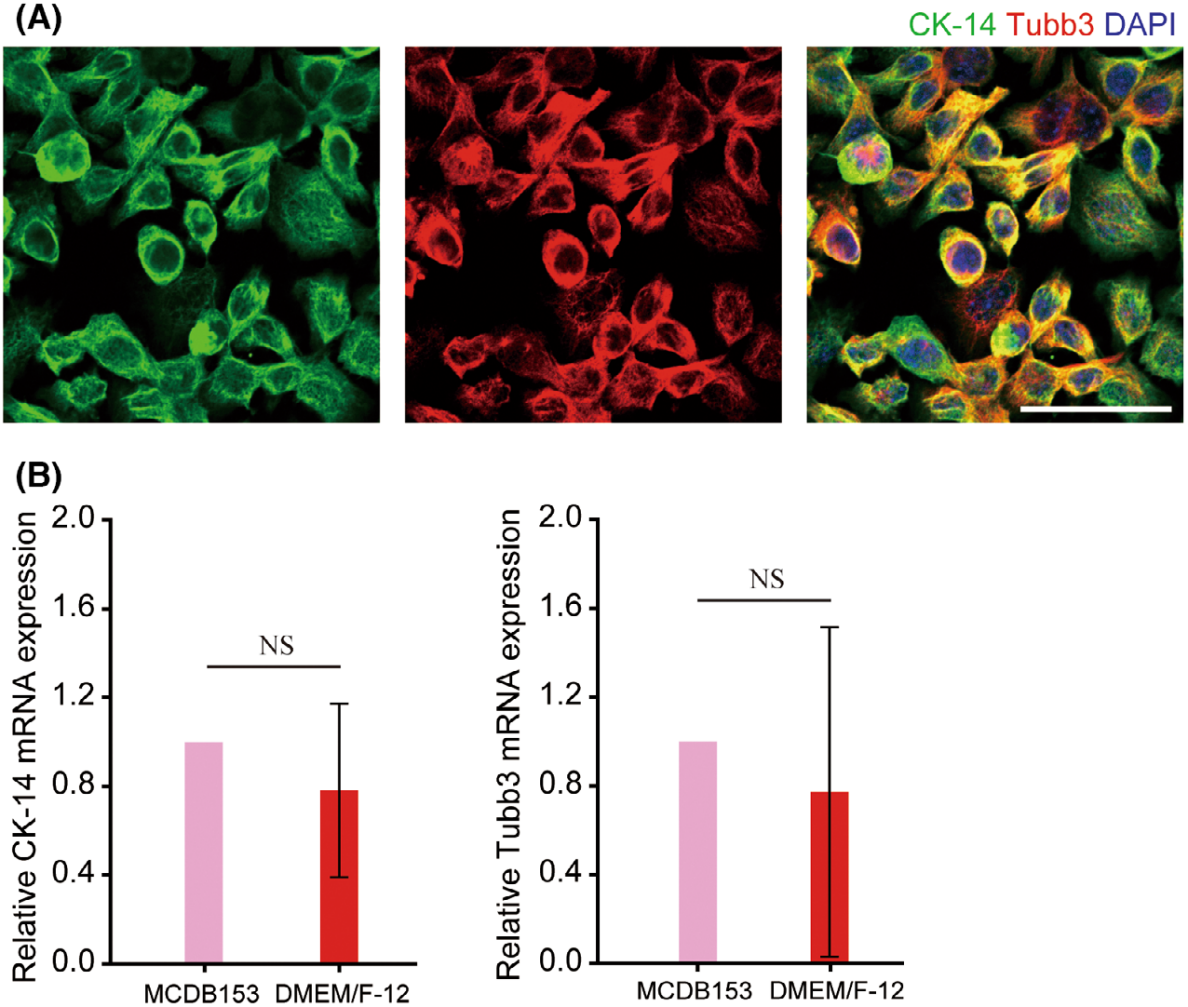
Olfactory basal cell‐specific expression pattern. (A) Immunofluorescence images of DBC1.2 cells cultured in DMEM/F‐12 medium showing the expression of Cytokeratin 14 (green) and class III β‐tubulin (red). Scale bar = 50 μm. (B) mRNA expressions of Cytokeratin 14 (CK‐14) and class III β‐tubulin (Tubb3) under different culture media. Bars represent the standard deviation (SD) of data. Statistical analyses were performed using Student's *t*‐test. *n* = 3 per group.

**Fig. 2:**
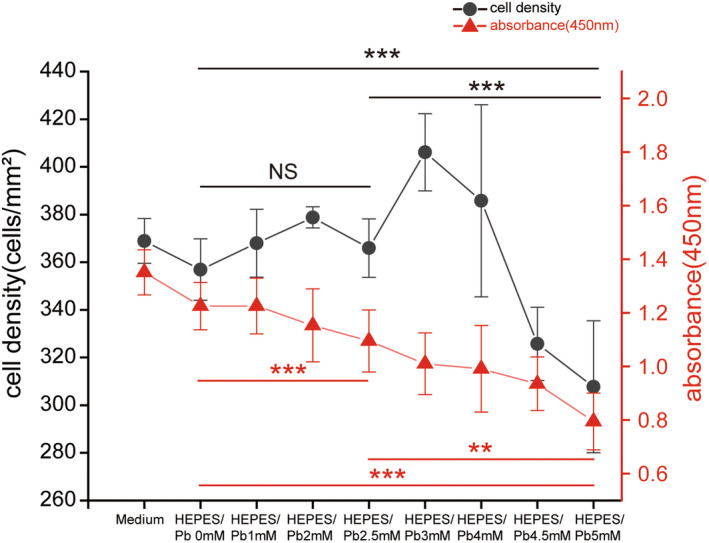
Pb‐exposed cytotoxicity evaluated with cell counting and the formazan test in DBC1.2 cells. Pb‐exposed cytotoxicity evaluation is shown in a double *y*‐axis graph. The cell count 1 day after 1 h of Pb exposure (left *y*‐axis, black) is shown as cell density. Cell density shows an irregular decline tendency with significant differences between the different concentrations of Pb solution. Cell metabolic activity measured with the WST‐8 formazan assay 1 day after Pb exposure is shown as optical density (O.D.) (right *y*‐axis, red). The higher values indicate higher metabolic activity. The optical density presents a relatively regular Pb dose‐dependent decline tendency, and there is a significant difference among different groups. Bars represent the SD of data. Statistical analyses were performed using one‐way ANOVA. *n* = 5 per group. **P < 0.05, ***P < 0.001.

**Fig. 3:**
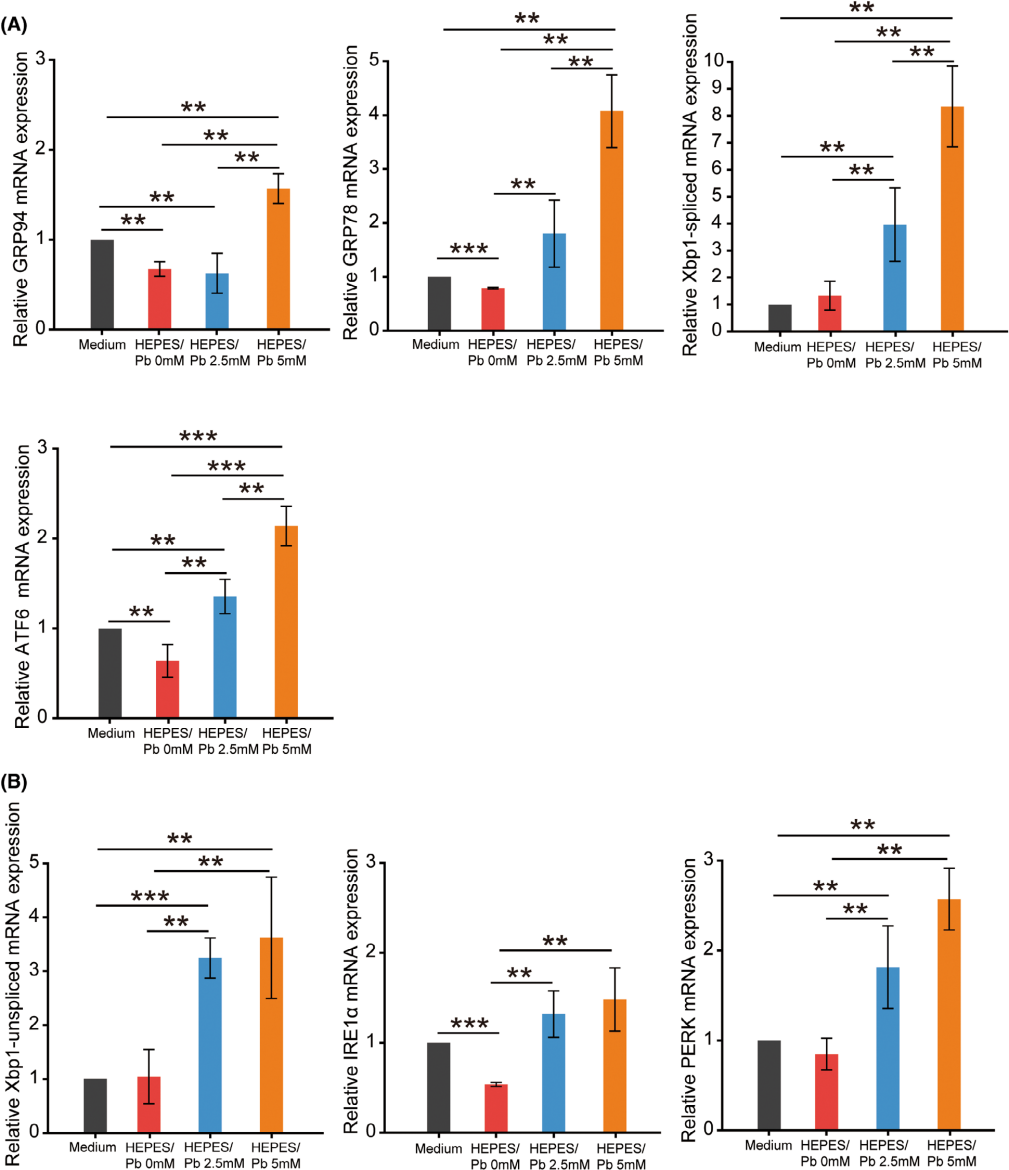
Pb exposure‐induced ER stress in DBC1.2 cells. The mRNA expression levels of ER stress‐related molecules under DMEM/F‐12 medium, HEPES buffer, HEPES/Pb 2.5 mm solution, and HEPES/Pb 5 mm solution. (A) The mRNA expression levels of GRP94, GRP78, Xbp1‐spliced, and ATF6 were significantly higher after Pb exposure and show a dose‐dependent increase. (B) The mRNA expression levels of Xbp1‐unspliced, IRE1α, and PERK increased significantly after Pb exposure, but there was no further statistical increase under 5 mm Pb exposure relative to 2.5 mm exposure. Bars represent the SD of data. Statistical analyses were performed using one‐way ANOVA. *n* = 3 per group. **P < 0.05, ***P < 0.001.

**Fig. 4:**
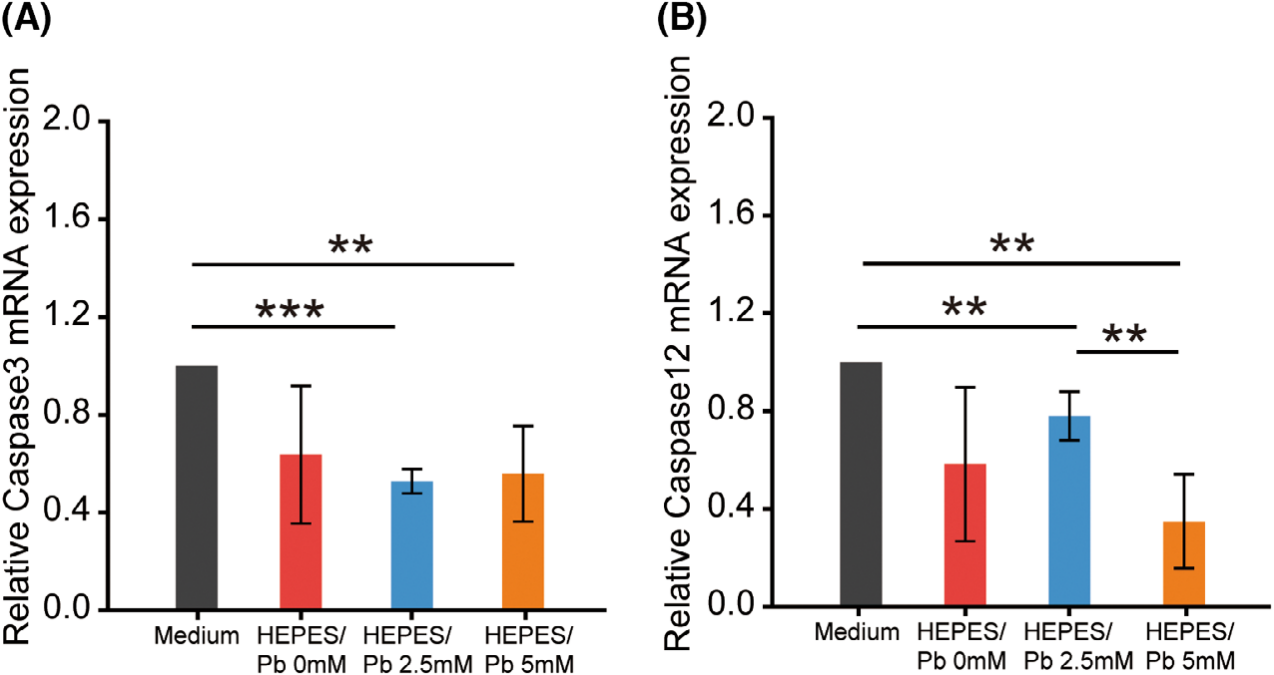
Pb exposure did not induce apoptosis in DBC 1.2 cells. The mRNA expression levels of Caspase 3 and Caspase 12 under DMEM/F‐12 medium, HEPES buffer, HEPES/Pb 2.5 mm solution, and HEPES/Pb 5 mm solution. The mRNA expression of Caspase 3 (A) and Caspase 12 (B) did not increase significantly after Pb exposure. Bars represent the SD of data. Statistical analyses were performed using one‐way ANOVA. *n* = 3 per group. **P < 0.05, ***P < 0.001.

**Fig. 5:**
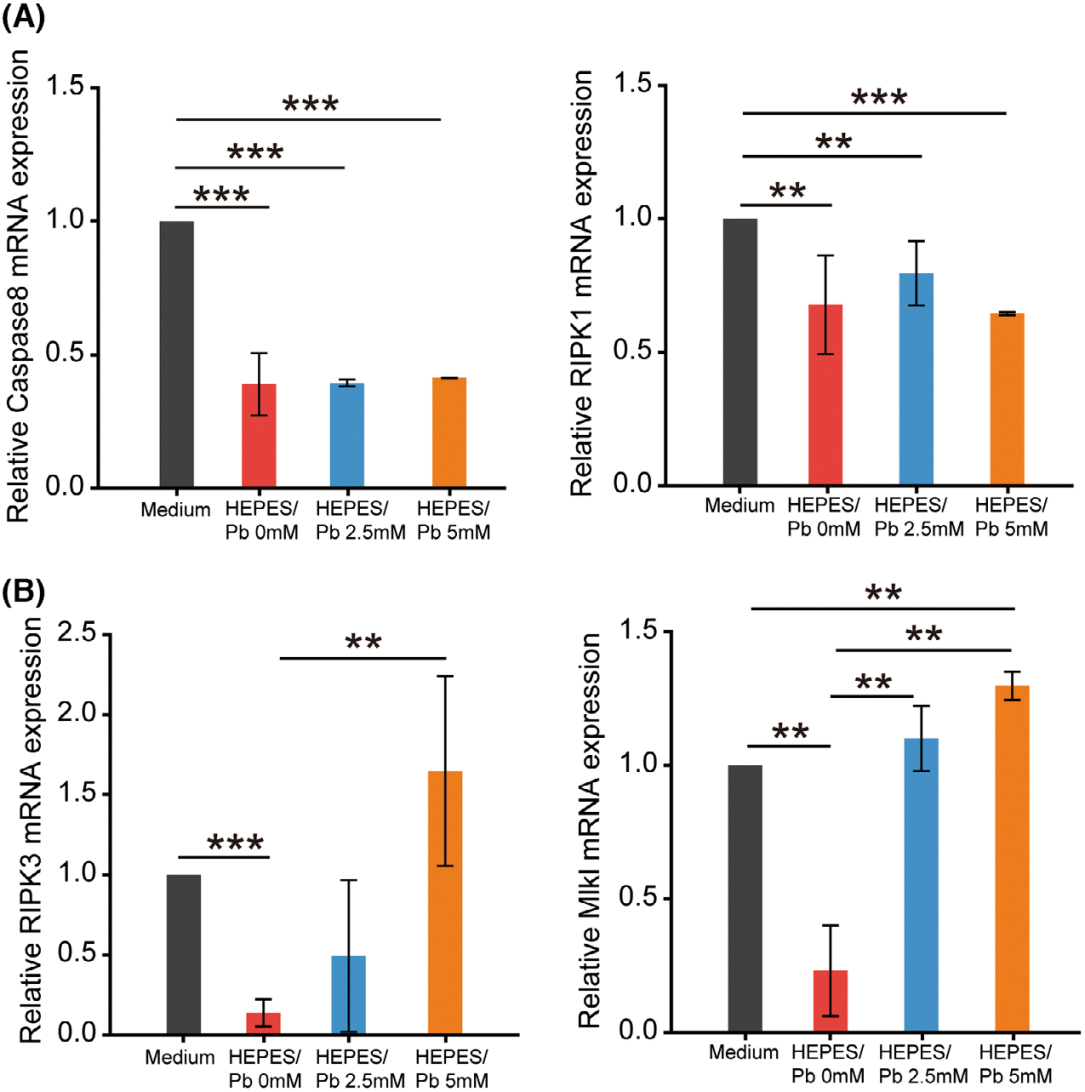
Pb exposure‐induced cell necroptosis in DBC1.2 cells. The mRNA expression levels of cell necroptosis markers under DMEM/F‐12 medium, HEPES buffer, HEPES/Pb 2.5 mm solution, and HEPES/Pb 5 mm solution. (A) The mRNA expression of Caspase 8 (left) and RIPK1 (right) did not increase after Pb exposure. (B) The mRNA expression levels of RIPK3 (left) and MLKL (right) increased significantly after Pb exposure. Bars represent the SD of data. Statistical analyses were performed using one‐way ANOVA. *n* = 3 per group. **P < 0.05, ***P < 0.001.

All data were presented as mean ± standard deviation (SD) and were statistically evaluated in the origin pro software (Origin Lab Corporation, Northampton, MA, USA) and jmp statistical discovery software (SAS Institute Japan, Tokyo, Japan). Data in Fig. 1C were analyzed with the Student's *t*‐test, and one‐way ANOVA was used to make multiple comparisons between groups in Figs 2, 3, 4, 5. A value of *P* < 0.05 was considered significant.

## Results

### DBC1.2 cell lines showed a cytokeratin‐14‐positive and class III β‐tubulin‐positive phenotype identical to olfactory basal cells

The olfactory horizontal basal cells have a multipotent progenitor phenotype [39], and Cytokeratin 14 (CK14)‐positive and class III β‐tubulin (TUBB3)‐positive cells are typical in basal cells [40]. Because the cell proliferation speed is very slow in the original medium formulation, the cells were cultured in DMEM/F‐12 supplemented with 10% FBS to promote cell proliferation, and then the phenotype changes under DMEM/F‐12 with FBS were analyzed. The expression of CK14 and TUBB3 were also positive under the modified culture medium condition (Fig. 1) and did not have significant changes in expression levels, which indicated that the phenotype under the original formulation was also observed in the cells under FBS‐supplemented medium. In the later analysis, the cells were cultured under DMEM/F‐12 with FBS.

### Pb exposure induced dose‐dependent cytotoxicity in DBC1.2 cells

The choice of buffer is important when investigating the effects of Pb on biological systems as negative findings may result from the use of inappropriate buffers [34]. HEPES buffer was adopted in this study because Pb does not precipitate in the working concentration of HEPES buffer. The DBC1.2 cells were exposed to Pb for 1 h, and the toxicity was analyzed using a WST‐8 formazan assay 1 day after Pb exposure. Lead exposure induced dose‐dependent cell toxicity and the 2.5 mm exposure induced approximately half decrease, and the cell number also showed an irregular dose‐dependent decrease after Pb exposure for 1 h (Fig. 2). At 2.5 mm, the cell metabolism was damaged without a significant decrease in cell numbers, while at 5.0 mm, both the cell metabolism and the cell numbers decreased. Based on these results, Pb exposure with concentrations of 2.5 or 5.0 mm for 1 h was utilized in the later analysis.

### Pb exposure induced ER stress in DBC1.2 cells

The changes in ER stress‐related genes after Pb exposure were analyzed with quantitative PCR. The changes in the expression of GRP78 and GRP94 with ER stress showed a significant increase after Pb exposure, and IRE1α/XBP1, PERK, and ATF6 pathways were all increased significantly after 5 mm Pb exposure (Fig. 3). The increase was also significant after 2.5 mm Pb exposure except for GRP94, which indicated that the 2.5 mm concentration was not enough to completely drive the ER stress pathway. Among several axes of ER stress pathways, the XBP1‐spliced form showed the most evident increase after Pb exposure at a concentration of 5.0 mm, which reflects the dose‐dependent cytotoxicity of Pb exposure. The expressions of GRP94, GRP78, ATF6, and IRE1α decreased significantly in HEPES buffer compared with normal medium, which indicated the partial protective effect of HEPES in the ER stress pathway.

### Pb exposure induced necroptosis in DBC1.2 cells, whereas apoptosis induction was not evident

To analyze the downstream pathway of ER stress induced by Pb exposure, the expressions of apoptosis markers were analyzed. The expressions of both Caspase 3 and Caspase 12 did not show significant increases after Pb exposure (Fig. 4). The expression of Caspase 12 decreased significantly in 5.0 mm Pb exposure compared with 2.5 mm, which indicated the stimulation of another cell death pathway other than apoptosis. The markers of necroptosis were also analyzed in DBC1.2 cells after Pb exposure, and the expression of RIPK3 and MLKL increased significantly after Pb exposure among Caspase 8, RIPK1, RIPK3, and MLKL (Fig. 5). These results indicated that Pb‐induced necroptosis rather than apoptosis took place in DBC1.2 cells, whereas Pb exposure at a concentration of 5.0 mm did not fully stimulate the necroptosis pathway. The expression of Caspase 8, RIPK1, RIPK3, and MLKL decreased significantly in HEPES buffer compared with normal medium, which indicated the protective effect of HEPES in both the apoptosis and necroptosis pathways.

## Discussion

In this study, Pb exposure induced dose‐dependent cell toxicity, and increases in stimulation of the ER stress pathway and partial stimulation of the necroptosis pathway were observed in the olfactory cells after 5.0 mm Pb exposure, whereas the apoptosis pathway was not stimulated. In addition, the HEPES buffer showed a partially protective effect in terms of ER stress, apoptosis, and necroptosis.

Increases in the stimulation of ER stress pathways were observed after Pb exposure, which was consistent with a previous study of ER stress showing that GRP78 as well as ATF4, ATF6, phospho‐IRE1, and XBP1 were increased in rat liver after Pb exposure [41].

The apoptosis pathway was not induced with Pb exposure in contrast to other heavy metals in DBC1.2 cells, which may reflect the fact that each heavy metal stimulates a different molecular pathway. In arsenite‐induced toxicity, mitochondrial ROS, ER stress, and Nrf2 crosstalk were induced, which promoted the mitochondrial permeability transition (MPT) and the MPT‐dependent apoptosis or necrosis [42], whereas the selection of pathways in apoptosis or necrosis depends on the cell types or conditions [43]. Lead exposure‐induced apoptosis or necrosis is related to the mechanism of the infiltration of inflammatory cells [44], and high‐dose Pb exposure enhances the levels of Caspase 8, Caspase 9, and Bax in liver, kidney, and brain [3]. In the kidney of rats, Pb exposure induces apoptosis while puerarin can alleviate damage through antioxidant activity and the modulation of the PI3K/Akt/eNOS signaling pathway [45]. In the liver of rats, Pb exposure has a disruptive effect on the mitochondrial respiratory complexes and causes oxidative stress through MPT‐pore opening and cytochrome c release, which promotes cell death signaling pathways including apoptosis or necrosis [46]. The mechanism of cell death pathway selection needs further analysis including understanding of mitochondrial calcium homeostasis, MPT, and mitochondrial‐ER cross‐talks.

The buffer selection for Pb exposure is limited due to Pb precipitation. The number of previous studies analyzing Pb exposure is relatively small than other heavy metals such as As, Cd or Hg, because a stable buffer that can hold the ionic state of Pb is limited. In addition, toxicity pathways may differ between organic and inorganic lead compounds. In this study, HEPES buffer was utilized in Pb exposure based on the results of a previous study [34], and the decreases in the expressions of several apoptosis‐ or necroptosis‐related genes were observed after being cultured with HEPES buffer compared with normal culture medium. HEPES has several biological activities [47], and it is possible that HEPES modulates the selection of cell death pathways. The previously reported biological effects of HEPES are the inhibition of taurine uptake in glial cells [48], the modulation of energy‐dependent efflux and uptake processes [49], the modulation of whole‐cell currents in cultured chemoreceptors of the rat carotid body [50], and the promotion of protein transfection. HEPES also has potential for a myriad of clinical applications [51], including the inhibition of the conversion of prion proteins in cell culture [52], the increase of glucocerebrosidase (GCase) activity in Gaucher disease‐patient derived fibroblasts [53], and the protection of myocardial tissue in immature myocardial ischemia–reperfusion injury [54], which are all cell‐protective functions. The mechanism of these molecular biological effects may include lysosome biogenesis with regard to the microphthalmia (MiT/TFE) family members, transcription factor EB (TFEB), and transcription factor E3 (TFE3) regulatory mechanisms that control the cytosolic retention [55]. The results of Pb exposure using HEPES buffers in this study may indicate expressions under partially protected conditions.

The stimulation of the necroptosis pathway by Pb exposure was observed as increases of RIP3K and MLKL, whereas RIP1K did not increase. RIP3K regulates necrosis‐specific RIP1K phosphorylation [56], and the kinase activity of RIP3 is essential for necrosis execution [57], which indicates the downstream and feedback functions of RIP3K in necroptosis. Caspase 8 did not increase, so the results in this study may reflect the protective function of HEPES and may represent a nontypical type of necroptosis. The evaluation of Pb exposure in buffers other than HEPES buffer is difficult due to precipitation issues, and the results in this study may differ from that of other reports evaluating heavy metal‐induced cell dysfunction.

## Conclusions

Lead exposure induced dose‐dependent cytotoxicity through ER stress and the necroptosis pathway in DBC1.2 cells, whereas the apoptosis pathway was not stimulated significantly. The necroptosis pathway plays a crucial rule in Pb exposure‐induced cytotoxicity in olfactory cells.

## Conflict of interest

The authors declare no conflict of interest.

## Author contributions

TK and SK conceived this study. TK, SK, and TY contributed to supervising experimental design. BH conducted the experiment. BH and TK performed collection of the data and statistical analysis. TY contributed to the final approval of the manuscript. All authors contributed to the writing of the manuscript.

## Data Availability

Data are available from the corresponding author upon a reasonable request.
