# Corrigendum to “Endoplasmic reticulum stress associated with lead (Pb)‐induced olfactory epithelium toxicity in an olfactory dark basal cell line” https://doi.org/10.1002/2211‐5463.13714

**DOI:** 10.1002/2211-5463.13759

**Published:** 2024-01-10

The Acknowledgements in the original article were incomplete. The full Acknowledgements section should read as follows:

This work was supported by JSPS KAKENHI grant nos. 21K16853 and 20K09705 and The Tokyo Society of Medical Sciences. We would like to thank Ayano Mitsui and Atsuko Tsuyuzaki for technical assistance. We would to like to thank Laura Mezey for reviewing the English language in this manuscript.

